# Consensus Statement der Österreichischen Gesellschaften für Rheumatologie und Rehabilitation, Pneumologie, Infektiologie, Dermatologie und Gastroenterologie zum Umgang mit latenter Tuberkulose bei Therapien mit biologischen oder „targeted synthetic“ DMARDs („*d*isease *m*odifying *a*nti*r*heumatic *d*rugs“)

**DOI:** 10.1007/s00393-022-01274-6

**Published:** 2022-11-07

**Authors:** Eva Rath, Michael Bonelli, Christina Duftner, Johann Gruber, Peter Mandl, Florentine Moazedi-Furst, Herwig Pieringer, Rudolf Puchner, Holger Flick, Helmut J. F. Salzer, Günter Weiss, Stefan Winkler, Hans Skvara, Alexander Moschen, Harald Hofer, Julia Feurstein, Judith Sautner

**Affiliations:** 1 Österreichische Gesellschaft für Rheumatologie und Rehabilitation (ÖGR), Wien, Österreich; 2 Österreichische Gesellschaft für Pulmologie (ÖGP), Wien, Österreich; 3 Österreichische Gesellschaft für Infektionskrankheiten und Tropenmedizin (ÖGIT), Kottingbrunn, Österreich; 4 Österreichische Gesellschaft für Dermatologie und Venerologie (ÖGDV), Wien, Österreich; 5 Österreichische Gesellschaft für Gastroenterologie und Hepatologie (ÖGGH), Wien, Österreich; 6 1. Medizinische Abteilung, Hanusch Krankenhaus, Wien, Österreich; 7 Universitätsklinik für Innere Medizin III, Klinische Abteilung für Rheumatologie, Medizinische Universität Wien, Wien, Österreich; 8 Universitätsklinik für Innere Medizin II, Department für Innere Medizin, Medizinische Universität Innsbruck/Tirol Kliniken, Innsbruck, Österreich; 9 Universitätsklinik für Innere Medizin, Klinische Abteilung für Rheumatologie und Immunologie, Medizinische Universität Graz, Graz, Österreich; 10 Klinik Diakonissen Linz, Linz, Österreich; 11 Ordination Wels, Wels, Österreich; 12 Universitätsklinik für Innere Medizin, Klinische Abteilung für Pulmonologie, Medizinische Universität Graz, Graz, Österreich; 13 Klinik für Lungenheilkunde, Kepler Universitätsklinikum Linz, Linz, Österreich; 14 Universitätsklinik für Innere Medizin I, Klinische Abteilung für Infektionen und Tropenmedizin, Medizinische Universität Wien, Wien, Österreich; 15 Abteilung für Dermatologie und Venerologie, Landesklinikum Wiener Neustadt, Wien, Österreich; 16 Klinik für Innere Medizin mit Schwerpunkt Gastroenterologie/Hepatologie, Kepler Universitätsklinikum Linz, Linz, Österreich; 17 Abteilung für Innere Medizin 1, Klinikum Wels-Grieskirchen, Wels, Österreich; 18 2. Medizinische Abteilung mit Schwerpunkt Rheumatologie, Karl Landsteiner Institut für klinische Rheumatologie, Landesklinikum Korneuburg-Stockerau, Landstr. 18, 2000 Stockerau, Österreich

**Keywords:** Screening, Präventivtherapie, Empfehlungen, Risikoreduktion, Medikamente, Screening, Preventive treatment, Recommendations, Risk reduction, Drugs

## Abstract

In diesem österreichweiten Consensus Statement sind die Empfehlungen zum Umgang mit latenter Tuberkulose bei Therapie mit biologischen und „targeted synthetic“ DMARDs zusammengefasst. Die wesentlichen Fragen bezüglich des Screenings und präventiver Therapie wurden von Expertinnen und Experten aus den Fachbereichen Rheumatologie, Pneumologie, Infektiologie, Dermatologie und Gastroenterologie auf Basis der vorhandenen Daten diskutiert und dann in einer Abstimmung zum gemeinsamen Konsens gebracht. Dabei wurde auch differenziert auf die unterschiedlichen Therapien eingegangen, und klare Empfehlungen wurden formuliert.

**Zusatzmaterial online:**

Die Online-Version dieses Beitrags (10.1007/s00393-022-01274-6) enthält weitere Tabellen und Abbildungen.

## Abstract (en)

This nationwide Austrian consensus statement summarizes the recommendations on the management of latent tuberculosis by treatment with biologic and targeted synthetic DMARDs. The essential questions with respect to screening and preventive treatment were discussed by experts from the disciplines of rheumatology, pneumology, infectious diseases, dermatology and gastroenterology, based on the available data, and then a joint consensus was formed by agreement. This involved a differentiated discussion on the various forms of treatment, and clear recommendations were formulated.

## Einleitung

Im Jahr 2011 erfolgte ein erster österreichweiter Consensus zum Umgang mit latenter Tuberkulose vor Therapie mit biologischen „disease modifying antirheumatic drugs“ (bDMARDs) [1]. Im Laufe der letzten 10 Jahre konnten nicht nur wesentliche neue Erkenntnisse hinsichtlich der Medikamentensicherheit gewonnen werden, sondern es wurde auch eine Vielzahl weiterer Präparate dieser Gruppe zugelassen. Aus diesen Gründen wurden in einer ExpertInnengruppe der ÖGR (Österreichische Gesellschaft für Rheumatologie und Rehabilitation), ÖGP (Österreichische Gesellschaft für Pneumologie), ÖGGH (Österreichische Gesellschaft für Gastroenterologie und Hepatologie), ÖGDV (Österreichische Gesellschaft für Dermatologie und Venerologie) und ÖGIT (Österreichische Gesellschaft für Infektionskrankheiten und Tropenmedizin) die Empfehlungen für die Diagnose und Therapie einer latenten Tuberkulose vor Beginn einer b/tsDMARD-Therapie neu erarbeitet und die Ergebnisse im Folgenden zusammengefasst.

### Tuberkuloseinzidenz und latente Tuberkulose

Trotz des weltweiten Rückgangs der Zahlen stellt die Tuberkulose auch im Jahr 2021 ein immenses globales Gesundheitsproblem dar. Allein im Jahr 2019 erkrankten laut WHO-Bericht 10 Mio. Menschen an dieser Infektionserkrankung, und 1,4 Mio. Menschen sind an den Folgen der Tuberkulose verstorben [2]. Das Auftreten der Erkrankung ist jedoch in den unterschiedlichen Regionen der Erde sehr ungleich verteilt, und somit sind die Inzidenzen von Land zu Land äußerst variabel. Fast die Hälfte der Erkrankten befindet sich in nur 30 Ländern, denen allesamt eine schlechte sozioökonomische Versorgung ihrer Einwohner gemein ist.

Im Vergleich dazu besteht in Österreich – wie in den meisten nord-, zentral- und westeuropäischen Ländern – eine sehr niedrige Tuberkuloseinzidenz mit 4,4 Fällen pro 100.000 Einwohnern bzw. 388 gemeldeten Tuberkulosefällen gesamt-österreichweit im Jahr 2020. Diese Zahl ist auch im Vergleich zu 2011 weiter gesunken [3] (ESM Abb. 1).

Im Gegensatz zur Verbreitung der manifesten Tuberkulose ist die Prävalenz der latenten Tuberkulose (LTBI) unklar. Als latente Tuberkulose bezeichnet man die asymptomatische Persistenz vitaler tuberkulöser Mykobakterien im Organismus nach einer Infektion. Die infizierte Person ist klinisch gesund und nicht ansteckend für ihre Umgebung. Als Zeichen einer stattgehabten Immunantwort auf den Erreger ist der Interferon-Gamma-Release-Test (IGRA) oder der Tuberkulinhauttest (TST) positiv, eine aktive Tuberkuloseerkrankung ist jedoch ausgeschlossen (unauffällige klinische Untersuchung und unauffälliges Thoraxröntgen) [4]. Verschiebt sich jedoch das Gleichgewicht zwischen immunologischer Kontrolle und bakterieller Aktivität zuungunsten der Immunität (wie z. B. unter immunsupprimierender Therapie), kann sich aus der LTBI eine aktive Tuberkulose entwickeln (Reaktivierung). Dabei kommt es zum Auftreten von klinischen Symptomen einer Tuberkuloseinfektion wie Fieber, Husten, Nachtschweiß oder Gewichtsverlust; auch fulminante Verläufe sind möglich.

### Wirkmechanismen der verschiedenen b-ts-DMARDs, deren Einfluss auf eine Tuberkuloseerkrankung und diesbezügliche Stellungnahmen in den Fachinformationen der Medikamente

#### Anti-CD20-Antikörper, Rituximab

Seit 1998 wird dieser gegen B‑Zellen gerichtete Antikörper in der Therapie von Lymphomen eingesetzt. Im Jahr 2006 erfolgte die Zulassung zur Behandlung der rheumatoiden Arthritis (RA). Des Weiteren wird Rituximab auch zur Behandlung ANCA-assoziierter Vaskulitis (Granulomatose mit Polyangiitis, mikroskopische Polyangiitis) sowie von Pemphigus vulgaris eingesetzt. In einer Vielzahl randomisierter kontrollierter Studien wie auch in Beobachtungsstudien und Registerdaten hat sich keinerlei Hinweis auf ein vermehrtes Auftreten von Tuberkulose gezeigt [5]. Auch in Tuberkulosehochinzidenzländern fand sich diesbezüglich kein Sicherheitsrisiko [6]. Die European Society of Clinical Microbiology and Infectious Diseases (ESCMID) verfasste 2018 hierzu eine klare Stellungnahme, die die Unbedenklichkeit hinsichtlich Tuberkulose unterstrichen hat [7]. Auch in der Fachinformation des Medikaments gibt es zu diesem Thema keine Warnung.

#### TNF-Blocker

Mit Beginn des Einsatzes des ersten TNF-Blockers (Infliximab) um die Jahrtausendwende fiel unter der laufenden Therapie bald ein gehäuftes Auftreten von Tuberkulosereaktivierungen auf, was in der Folge die Einführung eines routinemäßigen Screenings auf latente Tuberkulose vor Beginn der Therapie zur Folge hatte [8]. Durch Auswertung verschiedener Registerdaten zeigte sich, dass das Risiko, an einer aktiven Tuberkulose zu erkranken, unter Therapie in etwa 4fach erhöht ist [9]. Es fanden sich jedoch gewisse Unterschiede hinsichtlich der Tuberkuloseinzidenz zwischen den einzelnen TNF-Blockern. Das Fusionsprotein Etanercept zeigte wiederholt weniger Zahlen von Tuberkulosefällen und dürfte somit ein geringeres Risiko bergen als die anderen [10, 11].

Aufgrund der Lehre, die man aus der Markteinführungsphase der TNF-Blocker gezogen hatte, wurden die klinischen Studien der danach folgenden Biologikatherapien meist nur nach ausgeschlossener oder behandelter latenter Tuberkulose durchgeführt, was die Beurteilung des realen Risikos somit erschwert. Auch wurde in den Fachinformationen bei Wirkstoffen, die aus pathophysiologischer Sicht keinen wesentlichen Einfluss auf die Mykobakterienimmunantwort haben können und auch in Studien keinen Hinweis auf erhöhtes Tuberkuloserisiko zeigten, die Testung und Behandlung einer latenten Tuberkulose meist empfohlen.

#### Anti-IL-1, Anakinra, Canakinumab

Der erste Antikörper gegen IL‑1, einem zentralen Element der angeborenen Immunabwehr, kam 2002 auf den Markt. Die Bedeutung dieses Zytokins bei der Abwehr gegen Mykobakterien ist nicht ganz geklärt [12]. Im Rahmen der Zulassungsstudien wie auch in der weiteren Beobachtung zeigte sich jedoch nie ein Signal für ein vermehrtes Auftreten von Tuberkulose [13]. Zwar wurden die meisten Studien zu rheumatoider Arthritis hauptsächlich in Tuberkuloseniedriginzidenzländern durchgeführt, aber auch in Studien zur Behandlung des Morbus Behçet traten keine Tuberkulosefälle auf, obwohl hier eine erhöhte Inzidenz vorlag [14].

Nichtsdestotrotz findet sich in den Fachinformationen von Anakinra und Canakinumab die Empfehlung zu einem Screening auf LTBI.

#### Anti-CD80/86, Abatacept

Nach den Tuberkulosefällen rund um die Markteinführung der TNF-Blocker erfolgte in den Zulassungs- und Dosisfindungsstudien rund um den T‑Zell-Co-Stimulationshemmer Abatacept meist ein Screening nach LTBI. In den über 15 Jahren seit Markteinführung 2005 in den USA bzw. 2007 in Europa fand sich jedoch seither keinerlei Hinweis auf ein vermehrtes Auftreten von Tuberkulose. Zahlreiche Registerdaten und Beobachtungsstudien (teilweise aus Tuberkulosehochinzidenzländern) erbrachten keine oder ganz vereinzelte Fälle von Tuberkulose unter Therapie mit Abatacept [6]. Eine Analyse im Jahr 2018 von Daten verschiedener großer Abatacept-Studien mit insgesamt 21.335 Patientenjahren ergab 17 Fälle von Tuberkulose – allesamt in Hochrisikoländern und somit mit einem niedrigen Risiko zu bewerten [13, 15]. Diese Daten sprechen somit für ein äußerst geringes Risiko einer Tuberkuloseerkrankung unter Abatacept. Dennoch ist in der Fachinformation weiterhin das Screening auf LTBI empfohlen.

#### Anti-IL-6R, Tocilizumab, Sarilumab

Bei der Therapie mit Tocilizumab (IL-6-Rezeptor-Blockade) ging man nach den Ergebnissen bei TNF-Blockern ebenfalls äußerst vorsichtig vor. Die Medikamentengabe in den Zulassungsstudien wurde insgesamt nur nach Ausschluss einer latenten Tuberkulose durchgeführt, sodass das tatsächliche Tuberkuloserisiko unter dieser Therapie unerforscht blieb. Die Registerdaten, Beobachtungsstudien sowie auch einzelne Berichte von unbehandelter LTBI unter Tocilizumab ergaben zwar kein Signal hinsichtlich Tuberkulosegefahr dieser Medikamentenklasse, dennoch wurde angesichts des routinemäßigen Screenings vor Therapie die Gesamteinschätzung meist zugunsten einer präventiven Tuberkulosetherapie gemacht [6, 12, 13, 15]. Auch die Fachinformationen der Medikamente empfehlen das Screening und die Therapie einer LTBI.

#### Anti-IL-12/23, Ustekinumab

Die Blockade von IL-12 und IL-23 führt in der Theorie zu einer Beeinträchtigung der Immunantwort gegen Mykobakterien [16]. In Beobachtungen seit Markteinführung im Jahr 2009 zeigte sich bisher kein vermehrtes Auftreten von Tuberkulose unter Therapie mit Ustekinumab [6, 12, 15]. Es wurde jedoch ebenso wie bei den zuletzt genannten Therapien in Studien und auch im breiten Einsatz vor Beginn der Therapie auf LTBI gescreent, was die endgültige Beurteilung auch hier erschwert. Unterschiedliche Reviews beurteilen das Risiko für das Auftreten einer Tuberkulose als sehr niedrig. In der Fachinformation von Ustekinumab werden das Screening und die Therapie der LTBI empfohlen.

#### Anti-BLyS, Belimumab

Bei dieser gegen den B‑Lymphozyten stimulierenden Faktor (BLyS) gerichteten Antikörpertherapie kommt es zu einer Reduktion der Lebensdauer und Aktivität von B‑Lymphozyten. Die Therapie ist seit 2011 für die Behandlung von systemischem Lupus erythematodes (SLE) zugelassen. Weder besteht hier ein suspiziertes Tuberkuloserisiko, noch zeigte sich in Studien das Auftreten von Tuberkulosefällen [17]. In den meisten Studien wird das Wort Mykobakterien oder Tuberkulose gar nicht erwähnt. Auch laut Fachinformation von Belimumab ist ein Risiko bei latenter oder aktiver Tuberkulose unbekannt.

#### PDE4-Inhibitor (Phosphodiesterase-4-Inhibitor), Apremilast

Der Hemmer der Phosphodiesterase 4 war das erste Präparat, das der Gruppe der tsDMARDs („*t*argeted *s*ynthetic *D*isease *M*odifying *A*nti *R*heumatic *D*rug“) zugerechnet wird, die ihre Wirksamkeit durch Einfluss auf Signalwege innerhalb der Zellen entfalten. Apremilast, das 2015 auf den Markt kam, führt durch Hemmung von PDE4 zum Anstieg von cAMP (zyklisches Adenosinmonophosphat) und damit zu verminderter Bildung und Ausschüttung von Entzündungsmediatoren. Interessanterweise findet faktisch keine klinisch relevante Beeinträchtigung der Immunantwort hinsichtlich Infektionen bei Einsatz des Medikaments statt, weswegen es als sicher bei LTBI gesehen wird [6, 18]. In der Fachinformation steht keinerlei Hinweis zu Bedenken bezüglich Tuberkulose.

#### Anti-IL-17, Secukinumab, Ixekizumab, Brodalumab

Im selben Jahr kam auch der erste Vertreter dieser Medikamentenklasse auf den Markt. Das von T‑Helfer-Zellen gebildete IL-17 hat verschiedene Funktionen, insbesondere die Steigerung einer proinflammatorischen Immunantwort. Seine alleinige Blockade führt jedoch zu keiner klinisch relevanten Beeinflussung der allgemeinen Infektabwehr, lediglich zu einem etwas vermehrten Auftreten von *Candida*-Infektionen. Hinsichtlich einer verminderten Mykobakterienimmunantwort finden sich keinerlei Anzeichen [12, 19].

Auch gibt es in Untersuchungen aus Registerdaten und Beobachtungsstudien keine Hinweise für ein erhöhtes Risiko von Tuberkulose unter Anti-IL-17 [6, 15, 18]. Es existieren auch Fallberichte und Fallserien, wo Patienten mit LTBI ohne präventive Therapie eine Anti-IL-17-Therapie erhielten und kein einziger Fall einer Tuberkulosereaktivierung auftrat [20].

In den Zulassungsstudien der IL-17-Blocker wurde jedoch immer auf LTBI gescreent und diese ggf. behandelt, sodass keine evidenzbasierte Aussage zum definitiven Tuberkuloserisiko gemacht werden kann.

Aufgrund der zahlreichen indirekten Hinweise der Unbedenklichkeit bezüglich Tuberkuloserisiko wird in den Fachinformationen der verschiedenen IL-17-Blocker lediglich formuliert, dass ein Screening in Betracht oder Erwägung gezogen werden kann.

#### Anti-IL-23, Guselkumab, Risankizumab, Tildrakizumab

Eine Therapie gegen Anti-IL-23 ist seit 2017 zugelassen. Die Blockade dieses Zytokins führt zur Beeinflussung der Aktivität verschiedener Zellen des angeborenen und adaptiven Immunsystems, insbesondere T‑Zellen, Makrophagen und dendritische Zellen und hat somit theoretisch auch Einfluss auf die Mykobakterienimmunantwort [21]. In den Zulassungsstudien zu den Anti-IL-23-Therapien wurde immer auf LTBI gescreent und bei Vorliegen zumeist gegen LTBI behandelt. Bis dato kam es in klinischen und Real-World-Studien zu keiner Reaktivierung einer Tuberkulose [22].

In den Fachinformationen der Anti-IL-23-Therapien steht, dass auf LTBI untersucht und eine Therapie in Erwägung gezogen werden soll.

#### Januskinase(JAK)-Inhibitoren, Tofacitinib, Baricitinib, Upadacitinib, Filgotinib

Der erste Vertreter der JAK-Inhibitoren Tofacitinib ist seit 2017 zugelassen und erweiterte die Gruppe der tsDMARDs. Durch die Blockade der Januskinasen, die für die Signalwirkung verschiedener Zytokine von der Zelloberfläche in den Zellkern relevant sind, kommt es zur immunmodulierenden entzündungshemmenden Wirkung. Der Einfluss auf die Immunantwort gegen Mykobakterien wird ähnlich groß wie der von TNF-Blockern eingeschätzt [6, 23–25]. Es gibt jedoch naturgemäß keine Daten zu dieser Frage, weil ihr Einsatz von Anfang an nur nach Ausschluss oder Behandlung einer LTBI erfolgte. In der verlängerten Beobachtungszeit der Phase-II- und -III-Studien von Tofacitinib fanden sich 26 Tuberkulosefälle bei 5671 Patienten vorwiegend in Hochinzidenzländern, was auf ein überschaubares Risiko für eine Tuberkuloseinfektion bzw. Reaktivierung schließen lässt [23]. Die Fachinformationen der verschiedenen JAK-Inhibitoren sind unterschiedlich formuliert. Ein Screening soll gemacht werden; hinsichtlich präventiver Therapie geht die Empfehlung von „ist in Erwägung zu ziehen“ (Baricitinib, Upadacitinib) bis zu „LTBI sollte behandelt werden“ (Tofacitinib, Filgotinib).

#### „Receptor activator of NF-κB ligand“(RANKL)-Inhibitor, Denosumab

RANKL ist für die Umwandlung von Vorläuferzellen in die Knochen abbauenden Osteoklasten verantwortlich; seine Blockade vermindert daher den Knochenabbau. Ein zusätzlicher Einfluss auf das Immunsystem ist nicht bekannt, weswegen auch keinerlei Relevanz in der Mykobakterienimmunantwort vermutet wird. Seit 2010 ist das Medikament zugelassen und wird großflächig in der Behandlung der therapiepflichtigen Osteoporose eingesetzt. Das Medikament scheint somit hinsichtlich Tuberkulose unbedenklich; auch in der Fachinformation wird Tuberkulose nicht erwähnt [26]. Es gibt sogar einen Fallbericht eines erfolgreichen therapeutischen Einsatzes von Denosumab bei aktiver Tuberkulose mit Hyperkalzämie [27].

#### Sclerostin-Inhibitor, Romosozumab

Auch der Antikörper gegen Sclerostin (EMA Zulassung 2019) hat isoliert Einfluss auf den Knochenaufbau, aber keinerlei zusätzliche immunsuppressive Wirkung. Es wird daher kein Einfluss auf Tuberkulose vermutet; diese ist auch in der Fachinformation nicht erwähnt [28].

#### Integrin-Blocker, Vedolizumab

Der 2014 zugelassene Integrin-Blocker Vedolizumab verhindert das Andocken von aktivierten Lymphozyten im Darmgewebe [29]. Trotz dieser darmspezifischen Wirkung und bis dato keinen Berichten von Tuberkuloseerkrankungen unter Therapie formuliert die Fachinformation des Medikaments, dass auf LTBI untersucht und diese ggf. behandelt werden muss.

#### Anti-IgE, Omalizumab

Der Antikörper gegen IgE ist seit 2005 zugelassen und wird zur Behandlung von allergischem Asthma, chronischer Rhinosinusitis mit Polypen und chronischer spontaner Urtikaria eingesetzt. Weder theoretisch noch in Anwendungsbeobachtungen besteht ein erhöhtes Risiko für Tuberkulose [12]. Auch in der Fachinformation gibt es keinen Hinweis dazu.

#### Anti-C5(R), Eculizumab, Ravulizumab, Avacopan

Antikörper gegen ein Protein des terminalen Aktivierungswegs des Komplementsystems sind mit einer Anfälligkeit für Meningokokkeninfektionen vergesellschaftet (Eculizumab, Ravulizumab). Für den Antikörper Avacopan gegen den Rezeptor von C5a gilt dies nicht [12, 30]. Bezüglich Mykobakterieninfektionen bestehen keine Hinweise auf ein erhöhtes Risiko; auch in den Fachinformationen gibt es dazu keine Bemerkung.

#### Anti-IL-5(R), Mepolizumab, Reslizumab, Benralizumab

Antikörper gegen IL‑5 bzw. IL-5R werden zur Behandlung von schwerem eosinophilem Asthma eingesetzt und haben keinen erwartbaren Einfluss auf die Mykobakterienimmunantwort. Auch in Studien hat sich diesbezüglich keinerlei Hinweis gezeigt [12]. Die Fachinformationen führen ebenfalls keine Bemerkung hinsichtlich Tuberkulose an.

#### Anti-IL-4R/Anti-IL-13R, Dupilumab

Auch für den Antikörper gegen die Rezeptoren für IL‑4 und IL-13 ist keine Beeinflussung der Immunantwort gegen Mykobakterien bekannt oder beobachtet worden [31]. In der Fachinformation gibt es ebenfalls keinen Hinweis dazu.

#### Anti-IFNAR1, Anifrolumab

Der humane Antikörper gegen die Untereinheit I des Typ-I-Interferon-Rezeptors wurde 2022 zur Behandlung des moderaten bis schweren SLE zugelassen. Interferon alpha scheint eine Rolle in der zellulären Immunantwort gegen Mykobakterien zu spielen. Es greift in die Balance zwischen Keimabwehr und allgemeiner Inflammationsreaktion ein. Ob die Blockade von Interferon alpha einen negativen Einfluss auf die Mykobakterienabwehr hat, ist jedoch noch nicht geklärt [32–34]. In den Phase-II- und -III-Studien von Anifrolumab war eine LTBI ein Ausschlusskriterium wie in vielen anderen Studien zu Biologikatherapien. Die gepoolten Studiendaten der TULIP I- und TULIP II-Studien ergaben 4 Fälle von LTBI (IGRA wurde positiv ohne klinische oder radiologische Zeichen einer Tuberkuloseinfektion). Bei 459 Patienten unter Anifrolumab trat kein Fall einer aktiven Tuberkuloseinfektion auf [35]. Laut Fachinformation soll bei Patienten mit unbehandelter LTBI eine Antituberkulose(Anti-TBC)-Therapie in Erwägung gezogen werden.

### Bestehende internationale Empfehlungen zu LTBI und b-/ts-DMARDs

Die Leitlinien zur Diagnostik und Therapie der LTBI unterscheiden sich voneinander nur unwesentlich [4, 36, 37]. Zur Diagnose der LTBI wird immer ein IGRA („interferon-gamma release assay“) und/oder Tuberkulinhauttest („tuberculosis skin test“ [TST]) empfohlen. Für die Therapie der LTBI stehen 4 Therapieschemata zur Verfügung: Isoniazid (INH) für 9 Monate, Rifampicin (RIF) für 4 Monate, INH kombiniert mit RIF für 3 Monate oder Rifapentin mit INH wöchentlich für 3 Monate. Die Dosierung wird jeweils mit 5 mg/kg Körpergewicht für INH (maximal 300 mg/Tag) bzw. 10 mg/kg Körpergewicht für RIF (maximal 600 mg/Tag) angegeben. Die Gewichtung der verschiedenen Schemata ist in den 3 genannten Publikationen etwas unterschiedlich, aber insgesamt werden die Therapien als gleichwertig betrachtet. Die Verfügbarkeit von Rifapentin und auch die Notwendigkeit der wöchentlichen Verabreichung sind in Österreich nicht gegeben.

Bezüglich des Managements der LTBI im Rahmen von b/ts-DMARD-Therapie finden sich keine eindeutigen Empfehlungen in den Publikationen der internationalen Fachgesellschaften. In den aktuellen Richtlinien des American College of Rheumatology (ACR) zur Behandlung der RA, die 2021 publiziert wurden, findet sich lediglich ein Verweis, bei nichttuberkulösen Mykobakterieninfektionen eher Abatacept vor anderen b/ts-DMARDs zu verwenden, aber kein Hinweis auf den Umgang mit LTBI [38]. Die ACR-Richtlinien von 2015 empfehlen ein Screening mit IGRA oder TST sowie ggf. die präventive Tuberkulosetherapie vor Biologika- und Tofacitinib-Therapie [39]. In den rezenten Empfehlungen zum Management der RA der Europäischen Rheumagesellschaft EULAR (European Alliance of Associations for Rheumatology) wird das Vorgehen zur Behandlung der LTBI nicht eigens abgehandelt [40]. In den Richtlinien von 2013 wird bei LTBI und Kontraindikation gegen Chemoprophylaxe eine Rituximab-Therapie empfohlen [41]. Die Britische Gesellschaft für Rheumatologie (BSR) publizierte 2019 „biologic DMARD safety guidelines in inflammatory arthritis“ [42]. Es wird darin eine Chemoprophylaxe vor Biologikatherapie empfohlen, dabei aber betont, dass die Wahrscheinlichkeit einer Tuberkulosereaktivierung unter Rituximab und Abatacept sehr niedrig scheint. Zum Screening wird in den BSR-Guidelines die Kombination aus Thoraxröntgen und IGRA oder TST empfohlen. In den deutschen S2K-Leitlinien zur Tuberkulose im Erwachsenenalter von 2017 ist ein Kapitel der LTBI bei TNF-Inhibitoren und anderen Biologika gewidmet [37]. Hierin wird auf die SAFEBIO-Studie verwiesen [13], wo sich ein niedriges bis kein Tuberkuloserisiko für Rituximab, Abatacept, Tocilizumab, Ustekinumab und Anakinra findet. Als Screeningmethode werden IGRA und/oder TST empfohlen. In der S3-Leitlinie zur Behandlung der Psoriasis aus dem Jahr 2021 ist ein Kapitel dem Umgang mit LTBI gewidmet. Zwar werden ein Screening auf LTBI vor bDMARD-Therapie empfohlen (TNF-Blocker, Anti-IL-17, Anti-IL-12/23, Anti-IL-23) und auch die präventive Therapie einer LTBI, es wird jedoch hervorgehoben, dass unter Anti-IL-17 und Anti-IL-23 kein Hinweis auf ein erhöhtes Risiko einer Reaktivierung bekannt ist [43].

## Methoden

Die Auswahl der ExpertInnen spiegelt einen breiten Querschnitt wider sowohl durch die österreichische rheumatologische Landschaft mit 8 RheumatologInnen (universitär, außeruniversitär, niedergelassen, Mitglieder des Vorstands der Österreichischen Gesellschaft für Rheumatologie und Rehabilitation) als auch 2 Infektiologen, 2 Pulmologen, 1 Dermatologen sowie 1 Gastroenterologen. Zusätzlich erfolgten Unterstützung in der Schriftführung und Organisation und beratende Unterstützung in hepatologischen Fragen.

Nach einer ausführlichen Literatursuche durch ein Mitglied zu Inzidenz, Auftreten und Therapie von Tuberkulose unter diversen b/ts-DMARDS sowie Durchsicht von Leitlinien anderer Länder und der Fachinformationen der in Österreich zugelassenen Medikamente erfolgten eine schriftliche Information an alle Consensus-TeilnehmerInnen sowie die Formulierung von 8 Fragen.

Im Rahmen eines ersten (virtuellen) Consensus-Treffens aller TeilnehmerInnen am 19.04.2021 erfolgten eine ausführliche Diskussion der Thematik sowie bereits einstimmige Konsensfindung zu einzelnen Punkten.

Bei einem zweiten (virtuellen) Treffen mit 8 TeilnehmerInnen wurden die noch offenen Punkte diskutiert, und das Ergebnis wurde dann im Anschluss noch in Einzelgesprächen bzw. Einzelschriftverkehr mit den zu diesem Termin verhinderten TeilnehmerInnen besprochen.

Im Anschluss wurden die insgesamt 37 Stellungnahmen formuliert und an alle TeilnehmerInnen verschickt. Jeder Punkt wurde von allen TeilnehmerInnen in einer Likert-Skala 1–5 („strongly agree, agree, neither agree nor disagree, disagree, strongly disagree“) abgestimmt. Dabei wurde bereits in allen Punkten ein > 75 %ige Zustimmung („strongly agree or agree“) erreicht. In der weiteren Folge und nach Aussendung des Ergebnisses an alle TeilnehmerInnen (modifizierte Delphi-Technik) erfolgten noch eine teils schriftliche teils mündliche Diskussion und eine weitere Abstimmungsrunde, die dann das endgültige Abstimmungsergebnis brachte.

Nach Niederschrift der Statements in diesem Artikel wurde das Schriftstück an alle Teilnehmer zur Korrektur ausgesendet und schlussendlich nach Einbringen aller Kommentare und Zustimmung aller Teilnehmer und aller Fachgesellschaften zur Publikation eingereicht.

## Ergebnisse des Consensus

### Wann soll auf latente Tuberkulose gescreent werden?

- Vor Beginn einer bDMARD- oder tsDMARD-Therapie, bei der eine präventive Tuberkulosetherapie notwendig wäre, muss auf LTBI untersucht/gescreent werden (s. Heatmap: rot, orange). – 100 % Zustimmung

**Figure 1:**
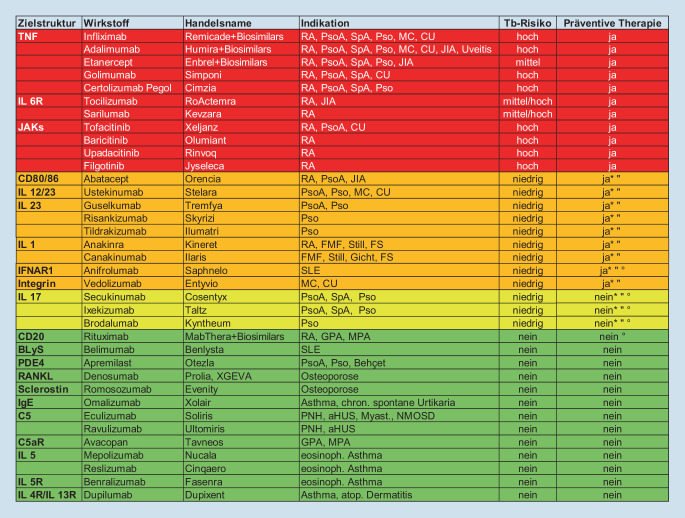

Die Entscheidung zum Screening auf LTBI erfolgt auf Basis der Therapie, die gestartet werden soll. Jene Medikamente, die kein erhöhtes Risiko für Reaktivierung einer LTBI bergen, benötigen demnach auch kein Screening. Aufgrund des Auftretens von Tuberkulosefällen in den Anfangsjahren der TNF-Blocker-Therapien erfolgte eine große Sensibilisierung für dieses Problem, weswegen in Zulassungsstudien für neuere bDMARD- und tsDMARD-Therapien die LTBI meist als Ausschlussgrund gehandhabt oder die LTBI präventiv therapiert wurde. Aus diesem Grund fehlen valide Daten zum wahren Risiko der Tuberkulosereaktivierung für die meisten Therapien, sodass die Einschätzung der ExpertInnenrunde aufgrund der vorhandenen publizierten Fallserien, nationalen Registerdaten, Post-Marketing-Beobachtung sowie der pharmakologisch-infektiologischen Bedeutung des jeweiligen Medikaments hinsichtlich Mykobakterienimmunantwort erfolgte. Auf Basis der vorhandenen Daten erfolgte eine Risikobewertung, die in Abb. 1 (Heatmap) dargestellt ist. Hierbei wurde eine Einstufung des Risikos durchgeführt, und zwar einerseits auf Basis des theoretischen pathophysiologischen Einflusses der Therapie und andererseits aufgrund der vorhandenen Daten zum Auftreten von Tuberkulose unter den verschiedenen Therapien. Zusätzlich flossen die Texte der Fachinformationen, die zum Teil ein Tuberkulosescreening unbedingt fordern, in die jeweilige Einstufung mit ein. Die Einteilung erfolgte in rot (hohes Risiko, präventive Therapie notwendig), orange (niedriges Risiko, präventive Therapie notwendig), gelb (niedriges Risiko, präventive Therapie nicht notwendig) und grün (kein Risiko, präventive Therapie nicht notwendig).

### Bei welchen bDMARD/tsDMARDs soll eine Therapie bei latenter Tuberkulose erfolgen?

Vor folgenden Medikamenten ist eine präventive Tuberkulosetherapie notwendig (Heatmap rot und orange):

- Anti-TNF – 100 % Zustimmung,
- Anti-IL-6R – 100 % Zustimmung,
- JAK-Inhibitoren – 100 % Zustimmung,
- Abatacept – 100 % Zustimmung,
- Anti-IL-12/23 – 100 % Zustimmung,
- Anti-IL-23 – 80 % Zustimmung,
- Anti-IL-1 – 80 % Zustimmung,
- Anti-IFNAR1 – 100 % Zustimmung,
- Vedolizumab – 66 % Zustimmung.

Vor folgenden Medikamenten ist eine präventive Tuberkulosetherapie *nicht* notwendig (Heatmap gelb und grün):

- Anti-IL-17 – 86 % Zustimmung,
- Anti-CD20 – 100 % Zustimmung,
- Anti-BLyS – 100 % Zustimmung,
- Apremilast – 100 % Zustimmung,
- RANKL-Inhibitor – 100 % Zustimmung,
- Sclerostin-Inhibitor – 100 % Zustimmung,
- Anti-IgE – 93 % Zustimmung,
- Anti-C5 – 93 % Zustimmung,
- Anti-IL-5 – 93 % Zustimmung,
- Anti-IL-4 – 93 % Zustimmung.

Die internationalen Richtlinien zur Behandlung von rheumatologischen Erkrankungen mit bDMARDs/tsDMARDs sprechen keine klaren Empfehlungen hinsichtlich einer präventiven LTBI-Therapie aus. Die Problematik wird entweder nicht angesprochen oder sehr allgemein formuliert und der Entscheidung der Behandlerin/des Behandlers auf Basis einer individuellen Risiko-Nutzen-Analyse überlassen.

Die vorhandenen Publikationen zu allen bDMARD/csDMARD-Therapien wurden hinsichtlich Tuberkuloserisikos durchsucht und bewertet, und jedes Mitglied stimmte bei jedem einzelnen bDMARD und tsDMARD hinsichtlich Notwendigkeit einer präventiven LTBI-Therapie ab. Bis auf 3 Medikamentengruppen gab es ein einstimmiges Ergebnis hinsichtlich Risikoeinschätzung und Notwendigkeit der präventiven Therapie (s. Ergebnis).

Bei Anti-IL‑1 waren 2 Teilnehmer gegen die Durchführung einer präventiven Therapie, und 1 Teilnehmer war unentschlossen. Die Entscheidung für eine präventive Therapie erfolgte hier v. a. aufgrund der Fachinformationstexte von Anakinra und Canakinumab, wo die Untersuchung auf LTBI empfohlen wird.

Bei Anti-IL-23-Therapie gab es 1 Stimme gegen die Durchführung der präventiven Tuberkulosetherapie und 2 unentschlossene Teilnehmer. Es gibt bei dieser Medikamentenklasse wenig theoretischen Einfluss auf die Mykobakterienimmunantwort und auch keine Berichte von Tuberkulosereaktivierungen in der Literatur. Da jedoch auch hier die Fachinformation die Untersuchung auf LTBI empfiehlt, erfolgte die Abstimmung mehrheitlich für die präventive LTBI-Therapie.

Anders fiel die Entscheidung bei Anti-IL-17-Therapie aus. Hier waren lediglich 1 Teilnehmer für die Durchführung der präventiven LTBI-Therapie und 1 Teilnehmer unentschlossen. Auch bei dieser Medikamentenklasse sprechen einerseits der fehlende Einfluss hinsichtlich Mykobakterienimmunantwort als auch fehlende Berichte von Tuberkulosereaktivierungen für die Ungefährlichkeit eines Einsatzes ohne präventive Therapie. Zusätzlich ist bei den Medikamenten dieser Klasse auch in den Fachinformationen die Untersuchung auf LTBI nicht unbedingt gefordert, sondern nur in Betracht zu ziehen. Diese Faktoren führten schlussendlich zum klaren Ergebnis der Abstimmung gegen die präventive Therapie. Die Behandlerin/der Behandler kann natürlich dennoch eine IGRA-Testung durchführen und bei positivem Ergebnis die Patienten über ein theoretisch niedriges, aber nicht gänzlich auszuschließendes Risiko aufklären.

Bei Vedolizumab, einem Medikament, das lediglich in der Gastroenterologie eingesetzt wird, waren 3 Teilnehmer unentschlossen und 2 gegen die Durchführung der präventiven Therapie. Da der gastroenterologische Vertreter klar für eine Therapie eintrat und auch in der Fachinformation sehr eindringlich die Therapie gefordert wird, erfolgte die Empfehlung somit – trotz nur 66 % Zustimmung – für eine Therapie.

ESM Tab. 1 zeigt eine Zusammenfassung der Fakten, die zur Entscheidung der Expertengruppe geführt haben.

### Wie soll auf LTBI gescreent werden?

- Das Screening auf LTBI umfasst eine Anamnese, einen IGRA-Test sowie ein Thoraxröntgen. – 100 % Zustimmung
- Bei nicht immunsupprimierten Personen sowie geplantem Niedrigrisikomedikament (s. Heatmap orange) kann auf ein Thoraxröntgen verzichtet werden. – 100 % Zustimmung
- IGRA-Befunde müssen immer gut dokumentiert werden. – 100 % Zustimmung
- Ein Tuberkulinhauttest (TST) bleibt Spezialfragestellungen vorbehalten. – 100 % Zustimmung

Das Screening auf LTBI umfasst eine ausführliche Anamnese zu möglicher Tuberkuloseexposition und sonstigen Risikofaktoren (stattgehabte Tuberkulose, Diabetes, Raucherstatus, Alkoholkonsum, Drogenkonsum, Mangelernährung, chronische Nierenerkrankung, Krebserkrankung etc.). Des Weiteren soll eine IGRA-Testung erfolgen, naturgemäß ist jedoch bei immunsupprimierten Personen eine gewisse Fehleranfälligkeit der Methode gegeben, wodurch es zu falsch negativen oder inkonklusiven Ergebnissen kommen kann. Insbesondere die Einnahme von Glukokortikoiden kann hier zu einem falsch negativen Ergebnis führen. Daher soll bei Immunsupprimierten auch immer ein Thoraxröntgen zum Ausschluss einer aktiven Tuberkulose erfolgen. Ein HRCT ist primär nicht gefordert.

Das Ergebnis und Datum der IGRA-Testung sollen gut dokumentiert werden, um zukünftigen BehandlerInnen Klarheit zu verschaffen.

Der Tuberkulinhauttest ist wegen seiner Beeinflussung durch die BCG-Impfung sowie Notwendigkeit von 2 Visiten innerhalb von 3 Tagen nur die zweite Wahl.

### Wie erfolgt die präventive Therapie der LTBI?

- Für die präventive LTBI-Therapie stehen folgende Behandlungsschemata zur Auswahl:
  - Rifampicin für 4 Monate (10 mg/kg, maximal 600 mg/Tag),
  - Isoniazid (INH) für 9 Monate (5 mg/kg, maximal 300 mg/Tag),
  - Kombination Rifampicin + INH für 3 Monate.
     – 100 % Zustimmung
- Komorbiditäten, Komedikationen, erwartbare Therapieadhärenz des Patienten sowie Verfügbarkeit der Medikamente sind bei der Auswahl zu berücksichtigen. – 100 % Zustimmung
- Die präventive Therapie soll gut dokumentiert werden, um zukünftigen BehandlerInnen Klarheit zu verschaffen. – 100 % Zustimmung
- Nach frühestens 4 Wochen präventiver Tuberkulosetherapie kann bei guter Verträglichkeit die Therapie mit einem bDMARD bzw. tsDMARD begonnen werden. – 100 % Zustimmung

Laut WHO Guidelines 2018, 2SK-Leitlinien 2017 und National Tuberculosis Controllers Association and CDC Guidelines 2020 stehen für die präventive Tuberkulosetherapie 4 Therapieschemata zur Auswahl (s. Tab. 1). Da Rifapentin in Österreich nicht erhältlich sowie die Notwendigkeit der 1‑mal wöchentlichen Einnahme nicht gegeben ist, wurde diese Therapieoption ausgeschieden. Die 3 übrigen Therapieschemata kommen in etwas unterschiedlicher Reihenfolge der Empfehlung in allen 3 Leitlinien vor, sodass wir uns zur gleichwertigen Empfehlung aller 3 Therapieschemata entschlossen haben. In der Diskussion der Teilnehmer fielen regionale Unterschiede des Einsatzes der Behandlungsschemata auf, wobei derzeit alle 3 in Österreich eingesetzt werden.

**Table 1:** 

Präventive Therapie der LTBI – 3 mögliche Schemata	
Isoniazid (INH)	Für 9 Monate
Rifampicin (RIF)	Für 4 Monate
INH + RIF	Für 3 Monate
*Dosierungen*	
INH	5 mg/kg Körpergewicht, maximal 300 mg/Tag
RIF	10 mg/kg Körpergewicht, maximal 600 mg/Tag

Die Vor- und Nachteile der einzelnen Therapieschemata betreffen das Nebenwirkungsspektrum der einzelnen Medikamente, das zusammen mit den Komorbiditäten und der Komedikation der Patienten in die Therapieentscheidung einfließen muss (s. ESM Tab 2). Auch ist bei einer Rifampicin-Therapie die Möglichkeit einer Cytochrom P450-assoziierten Wirksamkeitsreduktion von Glukokortikoiden zu beachten, weswegen eine Dosisanpassung der Glukokortikoiddosis notwendig sein kann. Doch auch die Therapiedauer und die Verfügbarkeit der Medikamente sind Faktoren, die die Entscheidung beeinflussen. Bei Einnahme von INH kann die gleichzeitige Gabe von Vitamin B_6_ (Pyridoxin) das Risiko für neurologische Nebenwirkungen reduzieren. Insbesondere in der Schwangerschaft und bei vorbestehendem Vitamin‑B_6_-Mangel oder Polyneuropathie ist auf eine Vitamin‑B_6_-Substitution zu achten.

Die Wichtigkeit der klaren Dokumentation der präventiven Therapie mit Angabe des Medikaments und der Therapiedauer wurde als wichtiger Einzelpunkt in den Consensus aufgenommen, um zukünftigen BehandlerInnen Klarheit zu verschaffen.

### Welche Kontrollen sollen während der Präventivtherapie erfolgen?

- Vor präventiver LTBI-Therapie sollen Anamnese, Patientenaufklärung sowie ein Basislabor (Blutbild, ALT, AST, GGT, AP, Bilirubin, Kreatinin) erfolgen. – 100 % Zustimmung
- Unter präventiver LTBI-Therapie soll nach 2 Wochen, danach 4‑wöchentlich die Bestimmung von Blutbild, ALT, AST, AP, GGT, Bilirubin und Kreatinin erfolgen. – 100 % Zustimmung
- Bei vorbestehender Lebererkrankung erfolgen individuelle Kontrollintervalle. – 100 % Zustimmung
- Besondere Vorsicht ist bei Kombination mit potenziell hepatotoxischen Substanzen geboten (Methotrexat, Leflunomid, Sulfasalazin). – 93 % Zustimmung
- Bei Transaminasenanstieg > 3fachen oberen Normwert sollen wöchentliche Kontrollen, bei > 5fachem Normwert soll ein Therapieabbruch erfolgen. – 100 % Zustimmung

Zu diesem Thema gibt es wenig Evidenz und keine klaren Richtlinien, sodass wir innerhalb des Consensus-Treffens, gefolgt von einer Diskussion mit einem Hepatologen, ein pragmatisches und trotzdem sicheres Vorgehen fanden, das die Zustimmung aller Teilnehmer erhielt. Hierbei wurde festgelegt, dass vorweg eine ausführliche Anamnese hinsichtlich vorbekannter Lebererkrankung, Alkoholkonsum und Komedikation (potenzielle Interaktionen) erfolgen soll. Des Weiteren ist eine Aufklärung der Patienten über Symptome einer potenziellen Leberschädigung (Oberbauchschmerzen, Ikterus, dunkler Harn, acholischer Stuhl, Juckreiz, Anorexie, Nausea) notwendig, und Ansprechpartner bzw. notwendige Maßnahmen sind zu besprechen. Auch soll über den negativen Einfluss von Alkoholkonsum bzw. Einnahme zusätzlicher Medikamente (Selbstmedikation, wie z. B. Paracetamol) aufgeklärt werden.

Die Kontrollintervalle der Laboruntersuchungen wurden mit anfangs 2‑ dann 4‑wöchentlich fixiert. Bei Anstieg der Werte sollen engere Intervalle gewählt werden und sind individuell zu gestalten. Jede neu aufgetretene Erhöhung der Leberwerte sollte auch hinsichtlich interkurrenter Lebererkrankungen evaluiert werden und bedeutet kürzere Kontrollintervalle. Insbesondere bei Anstieg der Transaminasen über den 3fachen oberen Normwert sollen wöchentliche Kontrollen erfolgen. Ab einem Anstieg über den 5fachen oberen Normwert soll die Therapie abgebrochen werden.

Das Herausstreichen von Methotrexat, Leflunomid und Sulfasalazin als eigener Punkt dient hierbei dazu, die Aufmerksamkeit der Behandlerin/des Behandlers auf diese im rheumatologischen Patientengut häufig verwendeten potenziell hepatotoxischen Medikamente zu lenken. Auch eine Therapiepause während Tuberkulosetherapie ist für Methotrexat zu überlegen.

Zusätzlich wurde in einem eigenen Punkt angeführt, dass bei vorbestehender Lebererkrankung die hier festgelegten Kontrollintervalle nicht als Richtlinie gelten, sondern vorsichtiger vorgegangen werden soll.

### Was macht man bei Unverträglichkeit der präventiven LTBI-Therapie?

- Bei Unverträglichkeit eines Tuberkulosemedikamentes soll das andere verfügbare Medikament versucht werden. – 100 % Zustimmung
- Bei Unverträglichkeit beider Tuberkulosemedikamente soll die Umstellung der rheumatologischen Basistherapie auf ein Niedrigrisikomedikament erfolgen (s. Heatmap grün bzw. gelb). – 100 % Zustimmung
- Die Nichttherapie der LTBI bei einer Basistherapie mit einem Medikament aus dem orange/roten Bereich der Heatmap erfordert einen mündlichen und schriftlichen informierten Consensus über die Nutzen-Risiko-Abwägung mit dem Patienten/der Patientin sowie engmaschige Kontrollen. – 93 % Zustimmung

Sollte sich eine Unverträglichkeit des Tuberkulosemedikamentes (INH, RIF) der ersten Wahl zeigen, kann auf ein Schema mit dem jeweils anderen Medikament gewechselt werden. Falls jedoch die Durchführbarkeit der präventiven Therapie aufgrund Unverträglichkeit beider Medikamente nicht möglich ist, soll eine rheumatologische Alternativtherapie mit einem Medikament mit niedrigem Tuberkuloserisiko erfolgen. Hierzu können Kombinationen von konventionellen DMARDs (csDMARDs) sowie bDMARD/tsDMARD mit niedrigem Tuberkuloserisiko (Heatmap: grün, gelb) herangezogen werden. Sollte es jedoch keinerlei alternative Behandlungsmöglichkeit für die Patientin/den Patienten geben, kann – unter ausführlicher Aufklärung über Nutzen und Risiken einer Therapie und gemeinsam mit der Patientin/dem Patienten – dennoch die Entscheidung zur Durchführung einer bDMARD/csDMARD-Therapie mit einem Medikament aus dem orangen (roten) Bereich der Heatmap erfolgen mit genauer schriftlicher Dokumentation dieser Aufklärung. In weiterer Folge ist unbedingt von beiden Seiten aufmerksam auf das Auftreten evtl. Symptome einer Mykobakterieninfektion zu achten, um diese ggf. sehr frühzeitig zu entdecken.

### Wie ist das Vorgehen nach einer abgeschlossenen Tuberkulosetherapie?

- Nach einer vollständig durchgeführten Therapie einer Tuberkulose oder einer LTBI muss im Verlauf (auch bei positivem IGRA-Befund) keine weitere Tuberkulosetherapie mehr durchgeführt werden (außer bei neuerlicher nachgewiesener Infektion). – 100 % Zustimmung

Sollte eine Patientin/ein Patient im Vorfeld eine Tuberkuloseerkrankung durchgemacht haben, die adäquat behandelt wurde (dokumentierte tuberkulostatische Kombinationstherapie über den erforderlichen Zeitraum), ist von der Abtötung sämtlicher Mykobakterien auszugehen und keine neuerliche präventive Tuberkulosetherapie mehr notwendig. Ein positiver IGRA spiegelt hier nicht eine latente Infektion wider, sondern ist ein Relikt der stattgehabten Infektion [44]. Im Zweifel bzw. bei mangelnder Dokumentation in der Anamnese ist individuell zu entscheiden und ggf. eine neuerliche tuberkulostatische Therapie einzuleiten.

Ebenso ist nach vollständig durchgeführter präventiver LTBI-Therapie mit der vollständigen Elimination der Mykobakterien zu rechnen. Ein weiterhin positiver IGRA ohne klinischen Hinweis auf eine neuerliche Tuberkuloseinfektion kann aber nur dann ignoriert werden, wenn die Adhärenz betreffend die stattgehabte tuberkulostatische Therapie gesichert ist und wenn eine neuerliche Infektion z. B. durch Reisen in ein Endemiegebiet ausgeschlossen werden kann.

### Was macht man bei inkonklusivem IGRA?

- Bei wiederholt inkonklusivem IGRA soll ein anderer Test (anderer IGRA, Tuberkulinhauttest) durchgeführt werden. – 100 % Zustimmung
- Bei weiterhin inkonklusivem Testergebnis kann – bei fehlendem Hinweis auf Tuberkulose im Thorax-CT – die bDMARD- bzw. tsDMARD-Therapie ohne präventive Tuberkulosetherapie gestartet werden. – 86 % Zustimmung

Das inkonklusive Ergebnis des IGRA kann durch Immundefizienz verursacht sein. Dies kann sowohl im Rahmen der Erkrankung als auch insbesondere durch eine immunsuppressive Therapie bedingt sein. Eine sehr relevante Rolle kommt hierbei einer Steroidtherapie zu, wo unbedingt versucht werden soll, die IGRA-Testung zu einem Zeitpunkt durchzuführen, wo keine oder zumindest nur niedrige Glukokortikoidmengen eingenommen werden.

Sollte es dennoch zu einem wiederholt inkonklusiven Testergebnis kommen, soll eine neuerliche Testung mit einem Alternativprodukt (IGRA eines anderen Herstellers, TST) gemacht werden.

Führt auch dies zu keinem eindeutigen Befund, können mithilfe einer CT der Lunge Hinweise auf eine aktive oder stattgehabte Tuberkulose ausgeschlossen werden. Bei fehlendem klinischem sowie Bildgebungshinweis (CT) auf eine Tuberkulose kann in einem Niedrigtuberkuloseinzidenzland wie Österreich die bDMARD/csDMARD-Therapie ohne präventive Tuberkulosetherapie begonnen werden.

### Wann ist die Wiederholung des IGRA notwendig?

- Die Wiederholung eines vormals negativen IGRA unter laufender bDMARD/tsDMARD-Therapie oder bei Wechsel der bDMARD/tsDMARD-Therapie ist nur bei klinischem Verdacht (z. B. Kontakt mit Tuberkulose, Reise in ein Endemiegebiet) notwendig. – 93 % Zustimmung

Während einer laufenden bDMARD/tsDMARD-Therapie ist keine routinemäßige Wiederholung des IGRA notwendig. Auch der Wechsel von einer bDMARD/tsDMARD-Therapie auf eine andere erfordert keine neuerliche Testung.

Natürlich können Testungen jederzeit durchgeführt werden, insbesondere jedoch bei relevantem Risiko für Exposition. Hierzu zählen Aufenthalte in Gebieten mit höherer Tuberkuloseinzidenz, Kontakt mit Personen mit Tuberkulose oder auch prekäre Wohnsituationen.

Eine Zusammenfassung der Consensus-Ergebnisse ist in der zusätzlichen Abb. 2 (ESM Abb. 2) dargestellt.

## Diskussion

Die österreichweite Vereinheitlichung des Vorgehens bei der Anwendung von bDMARD/tsDMARDs und latenter Tuberkulose war ein wesentlicher Faktor zur Erstellung dieses Consensus Statements. Es können naturgemäß nicht sämtliche Fragestellungen mit vollständiger Evidenz beantwortet werden, aber die Erfahrung mit den unterschiedlichen Therapien sowie die große Anzahl an Publikationen zu den meisten Therapien ermöglicht dennoch eine sehr gute Einschätzung des Risikos für Tuberkuloseerkrankungen. Zusätzlich besteht auch in Österreich als Tuberkuloseniedriginzidenzland eine weitere Risikoreduktion hinsichtlich Tuberkulose. Das führte dazu, dass die Einstufung von IL-17-Blockern in die Niedrigrisikogruppe und damit Verzicht auf ein Screening hinsichtlich LTBI erfolgte. Andere Medikamentenklassen (Heatmap orange) hätten zwar in der Einschätzung der TeilnehmerInnen teilweise ein ähnlich geringes Risiko für Tuberkulose, es wurde jedoch aufgrund der Fachinformationstexte schlussendlich doch zugunsten von Screening und präventiver LTBI-Therapie entschieden. Zukünftige kontrollierte, randomisierte Studien würden die Sicherheit bei dieser Fragestellung definitiv erhöhen.

Bezüglich Screeningmethoden soll dieser Consensus ebenfalls zu einer Vereinfachung und Vereinheitlichung der Herangehensweise innerhalb der Ärzteschaft führen.

Auch die Auswahl und Überwachung der Präventivtherapie, wie in diesem Consensus niedergeschrieben, haben das Ziel, den Einsatz der Therapien zu erleichtern und mögliche negative Auswirkungen für PatientInnen zu minimieren.

Auf Basis der hier angeführten Stellungnahmen soll allen in Österreich tätigen Ärztinnen und Ärzten der Umgang mit b/tsDMARDs erleichtert und sämtliche relevante Fragestellungen hinsichtlich latenter Tuberkulose mit dem derzeit verfügbaren Wissen bestmöglich beantwortet werden.

## Supplementary Information







