# Supplementary material for: National consensus statement by the Austrian Societies for Rheumatology, Pulmonology, Infectiology, Dermatology and Gastroenterology regarding the management of latent tuberculosis and the associated utilization of biologic and targeted synthetic DMARDS (disease modifying antirheumatic drugs)
Source: Z Rheumatol. 2022 Nov 7;82(2):163–74. [Article in German] doi: 10.1007/s00393-022-01274-6 (PMC9981509; doi:10.1007/s00393-022-01274-6)
Supplement: Supplementary file 1 [file 393_2022_1274_MOESM1_ESM.pdf]

Zusätzliche Abbildung 1

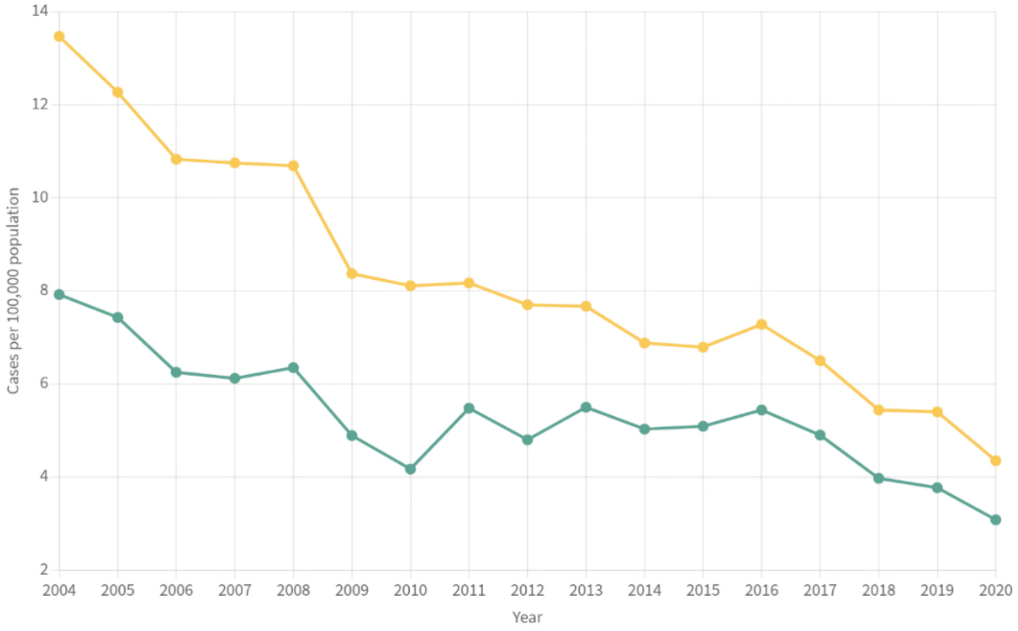

Gemeldete Tuberkulosefälle (gelb) und mikrobiologisch bestätigte Fälle des M. tuberculosis Komplex (blau) je 100.000 Bevölkerung in Österreich

Quelle: Homepage der Österreichische Agentur für Gesundheit und Ernährungssicherheit GmbH, abgerufen 2022-04-15: https://www.ages.at/en/mensch/krankheit/krankheitserreger-von-a-bis-z/tuberkulose?sword_list%5B0%5D=tuberculosis&no_cache=1
