# Supplementary material for: National consensus statement by the Austrian Societies for Rheumatology, Pulmonology, Infectiology, Dermatology and Gastroenterology regarding the management of latent tuberculosis and the associated utilization of biologic and targeted synthetic DMARDS (disease modifying antirheumatic drugs)
Source: Z Rheumatol. 2022 Nov 7;82(2):163–74. [Article in German] doi: 10.1007/s00393-022-01274-6 (PMC9981509; doi:10.1007/s00393-022-01274-6)
Supplement: Supplementary file 2 [file 393_2022_1274_MOESM2_ESM.pdf]

Zusätzliche Abbildung 2

Consensus statements und Angaben der Zustimmung der TeilnehmerInnen in %

Wann soll auf latente Tuberkulose gescreent werden?

Vor Beginn einer bDMARD- oder tsDMARD-Therapie, bei der eine präventive Tbc-Therapie notwendig wäre, muss auf latente Tbc untersucht/gescreent werden

(Heat-Map: rot, orange) 100%

Zielstruktur	Tb-Risiko	Screening	Präventive Therapie
**TNF**	hoch	ja	ja
**IL 6R**	mittel/hoch	ja	ja
**JAKs**	hoch	ja	ja
**CD80/86**	niedrig	ja	ja
**IL 12/23**	niedrig	ja	ja
**IL 23**	niedrig	ja	ja
**IL 1**	niedrig	ja	ja
**IFNRA1**	niedrig	ja	ja
**Integrin**	niedrig	ja	ja
**IL 17**	niedrig	nein	nein
**CD20**	nein	nein	nein
**BLyS**	nein	nein	nein
**PDE4**	nein	nein	nein
**RANKL**	nein	nein	nein
**Sklerostin**	nein	nein	nein
**IgE**	nein	nein	nein
**C5, C5aR**	nein	nein	nein
**IL 5, IL 5R**	nein	nein	nein
**IL 4R/IL 13R**	nein	nein	nein

Bei welchen bDMARD/tsDMARDs soll eine Therapie bei latenter Tuberkulose erfolgen?

Bei folgenden Medikamenten ist eine präventive Tuberkulose-Therapie notwendig (Heat-Map: rot, orange):

anti-TNF	100%
anti-IL6R	100%
JAK-Inhibitoren	100%
Abatacept	100%
anti-IL12/23	100%
anti-IL23	80%
anti-IL1	80%
anti-IFNAR1	100%
Vedolizumab	66%

Bei folgenden Medikamenten ist eine präventive Tuberkulose-Therapie NICHT notwendig (Heat-Map: gelb, grün):

Anti-IL17	86%
anti-CD20	100%
anti-BLyS	100%
Apremilas	100%
RANKL-Inhibitor	100%
Sklerostin-Inhibitor	100%
Anti-IgE	93%
Anti-C5	93%
Anti-IL5	93%
Anti-IL4	93%

Wie soll auf latente Tuberkulose gescreent werden

- Das Screening auf latente Tuberkulose umfasst eine
  Anamnese, einen IGRA-Test sowie ein Thorax-Röntgen. 100%
- Bei nicht immunsupprimierten Personen sowie geplantem Niedrig-Risiko-Medikament (Heat Map orange) kann
  auf ein Thorax-Röntgen verzichtet werden. 100%
- IGRA Befunde müssen immer gut dokumentiert werden. 100%
- Ein Tuberkulin Hauttest bleibt Spezialfragestellungen vorbehalten. 100%

Wie erfolgt die präventive Therapie der latenten Tuberkulose

- Für die präventive Tuberkulose-Therapie stehen folgende Behandlungsschemata zur Auswahl:
  -Rifampicin (RIF) für 4 Monate
  -Isoniazid (INH) für 9 Monate
  -Kombination Rifampicin + INH für 3 Monate 100%
- Co-Morbiditäten, Co-Medikationen, erwartbare Therapieadhärenz des Patienten, sowie Verfügbarkeit der Medikamente sind bei der
  Auswahl zu berücksichtigen. 100%
- Die präventive Therapie soll gut dokumentiert werden,
  um zukünftigen Behandlern Klarheit zu verschaffen 100%
- Nach frühestens 4 Wochen präventiver Tuberkulose-Therapie kann bei guter Verträglichkeit die Therapie mit einem
  bDMARD bzw. tsDMARD begonnen werden. 100%

Welche Kontrollen sollen während der Präventivtherapie erfolgen?

- Vor präventiver Tuberkulose-Therapie sollen Anamnese, Patientenaufklärung sowie ein Basislabor
  (Blutbild, ALT, AST, GGT, AP, Bilirubin, Kreatinin) erfolgen 100%
- Unter präventiver Tuberkulose-Therapie soll nach 2 Wochen, danach 4-wöchentlich die Bestimmung von Blutbild,
  ALT, AST, AP, GGT, Bilirubin und Kreatinin erfolgen. 100%
- Bei vorbestehender Lebererkrankung
  erfolgen individuelle Kontrollintervalle 100%
- Besondere Vorsicht ist bei der Kombination mit anderen potentiell hepatotoxischen Medikamenten geboten (Methotrexat, Leflunomid, Salazopyrin). 93%
- Bei Transaminasenanstieg >3fachen oberen Normwert sollen wöchentliche Kontrollen,
  bei >5fachen Normwert soll ein Therapieabbruch erfolgen. 100%

Was macht man bei Unverträglichkeit der Präventivtherapie?

- Bei Unverträglichkeit eines Tuberkulose-Medikamentes
  soll das andere verfügbare Medikament versucht werden. 100%
- Bei Unverträglichkeit beider Tuberkulose-Medikamente soll die Umstellung der rheumatologischen Basistherapie
  auf ein Niedrig-Risiko-Medikament erfolgen (Heat-Map: grün, gelb). 100%
- Die Nicht-Therapie der latenten Tuberkulose bei einer Basistherapie mit einem Medikament aus dem orange/roten Bereich der Heat-Map erfordert einen mündlichen und schriftlich dokumentierten Consensus über die Nutzen/Risikoabwägung
  mit dem Patienten/der Patientin sowie engmaschige Kontrollen. 93%

Wie ist das Vorgehen nach einer abgeschlossenen Tuberkulose-Therapie?

- Nach einer vollständig durchgeführten Therapie einer Tuberkulose oder einer latenten Tuberkulose muss im Verlauf (auch bei positivem IGRA Befund) keine weitere Tuberkulose-Therapie mehr durchgeführt werden (außer bei neuerlicher nachgewiesener Infektion). 100%

Was macht man bei inkonklusivem IGRA?

- Bei wiederholt inkonklusivem IGRA soll ein anderer Test (anderer IGRA, Tuberkulose-Hauttest) durchgeführt werden. 100%
- Bei weiterhin inkonklusivem Test-Ergebnis kann – bei fehlendem Hinweis auf Tuberkulose im CT-Thorax – die bDMARD bzw. tsDMARD-Therapie ohne präventive Tuberkulose-Therapie gestartet werden. 86%

Wann ist die Wiederholung des IGRA notwendig?

- Die Wiederholung eines vormals negativem IGRA unter laufender bDMARD/tsDMARD-Therapie oder bei Wechsel der bDMARD/tsDMARD-Therapie ist nur bei klinischem Verdacht (z.B. Kontakt mit Tuberkulose, Reise in ein Endemiegebiet) notwendig. 93%
