# Supplementary material for: National consensus statement by the Austrian Societies for Rheumatology, Pulmonology, Infectiology, Dermatology and Gastroenterology regarding the management of latent tuberculosis and the associated utilization of biologic and targeted synthetic DMARDS (disease modifying antirheumatic drugs)
Source: Z Rheumatol. 2022 Nov 7;82(2):163–74. [Article in German] doi: 10.1007/s00393-022-01274-6 (PMC9981509; doi:10.1007/s00393-022-01274-6)
Supplement: Supplementary file 3 [file 393_2022_1274_MOESM3_ESM.pdf]

Zusätzliche Tabelle 1

Zielstruktur	Tuberkulose-Screening notwendig-Expertenconsens	Theoretisches Tuberkuloserisiko	Evidenz für Tuberkuloserisiko	Laut Fachinformation Tuberkulose-Screening empfohlen
**TNF**	100%	ja	ja (8,9,10,11)	ja
**IL 6R**	100%	ja?	nein (6,12,13,15)	ja
**JAKs**	100%	ja?	nein (6,23,24,25)	ja
**CD80/86**	100%	nein?	nein (6,13,15)	ja
**IL 12/23**	100%	ja?	nein (6,12,15)	ja
**IL 23**	80%	ja?	nein (21,22)	ja
**IL 1**	80%	nicht klar	nein (13,14)	ja
**IFNAR1**	100%	nicht klar	nein (32,33,34,35)	nein
**Integrin**	66%	nein	nein (29)	ja
**IL 17**	7%	nein	nein (6,15,18,20)	nein
**CD20**	0%	nein	nein (5,6,7)	nein
**BLyS**	0%	nein	nein (17)	nein
**PDE4**	0%	ja?	nein (6,18)	nein
**RANKL**	0%	nein	nein (26,27)	nein
**Sklerostin**	0%	nein	nein (28)	nein
**IgE**	0%	nein	nein (12)	nein
**C5**	0%	nein	nein (12)	nein
**IL 5**	0%	nein	nein (12)	nein
**IL 4R/13R**	0%	nein	nein (31)	nein

Zusammenfassung der Fakten, die zur Expertenentscheidung geführt haben
