# Supplementary material for: National consensus statement by the Austrian Societies for Rheumatology, Pulmonology, Infectiology, Dermatology and Gastroenterology regarding the management of latent tuberculosis and the associated utilization of biologic and targeted synthetic DMARDS (disease modifying antirheumatic drugs)
Source: Z Rheumatol. 2022 Nov 7;82(2):163–74. [Article in German] doi: 10.1007/s00393-022-01274-6 (PMC9981509; doi:10.1007/s00393-022-01274-6)
Supplement: Supplementary file 4 [file 393_2022_1274_MOESM4_ESM.pdf]

Zusätzliche Tabelle 2:

Nebenwirkungen/Kontraindikationen/Interaktionen von INH und Rifampicin

	**Isoniazid**	**Rifampicin**
**Nebenwirkungen**	Polyneuritis (in Kombination mit Vitamin B6 Gabe deutlich reduziert), psychische Störungen, Verdauungsstörung, Hepatitis/lebertoxisch, Hautausschlag, Augenrötung	Müdigkeit, Benommenheit, Thrombozytopenie, Flu-like-Syndrom, Magen-Darm-Beschwerden, Hepatitis/lebertoxisch, Hautreaktionen, färbt Körperflüssigkeiten orangerot
**Kontraindikationen**	Überempfindlichkeit, periphere Neuritis, schwere Blutungsneigung, schwere Lebererkrankung	Überempfindlichkeit, schwere Leberfunktionsstörung, Porphyrie, gleichzeitige Anwendung mit Saquinavir/Ritonavir
**Interaktionen**	Barbiturate, Phenytoin, Carbamazepin, Primidon, Rifampicin, Valproinsäure, Paracetamol, Ketoconazol, Disulfiram, Alkohol, Antazida, Levodopa	CYP450-Induktor – Vielzahl an Interaktionen, insbesondere Antiepileptika, Benzodiazepine, Paracetamol, Azol-Antimykotika, antivirale Therapien, Antiarrhythmika,…
